# Feasibility of SMS to remind pregnant and breastfeeding women living with HIV to take antiretroviral treatment in Kilimanjaro region, Tanzania: a pilot study

**DOI:** 10.24248/eahrj.v4i2.637

**Published:** 2020-11-26

**Authors:** Kennedy M. Ngowi, Eusebious Maro, Rob E. Aarnoutse, Blandina T. Mmbaga, Mirjam A. G Sprangers, Peter Reiss, Pythia T. Nieuwkerk, I. Marion Sumari-de Boer

**Affiliations:** a Kilimanjaro Clinical Research Institute, Moshi, Tanzania, United Republic of; b Kilimanjaro Christian Medical Center, Moshi, Tanzania, United Republic of; c Kilimanjaro Christian Medical University College, Moshi, Tanzania, United Republic of; d Radboudumc, Radboud Institute for Health Sciences & Department of Pharmacy, Nijmegen, The Netherlands; e Amsterdam UMC, Location AMC, University of Amsterdam, Department of MedicalPsychology, Amsterdam, The Netherlands; f Amsterdam UMC, Location AMC, University of Amsterdam, Department of Global Health, and Amsterdam Institute for Global Health and Development, Amsterdam, The Netherlands; g HIV Monitoring Foundation, Amsterdam, the Netherlands; h Radboudumc, Department of Internal Medicine: infectious diseases, Nijmegen, the Netherlands

## Abstract

**Background:**

Pregnant and breastfeeding Women Living with HIV (WLHIV) often have difficulties in reaching adequate levels of adherence (>95%) to Antiretroviral treatment. “Forgetting” is the most commonly mentioned reason. Sending reminders via SMS is expected to improve adherence. We conducted a pilot study to investigate acceptability, user experience and technical feasibility of sending reminder-SMS to WLHIV.

**Methods:**

This was a 6-months observational pilot-study among WLHIV attending antenatal and postnatal care at Kilimanjaro Christian Medical Centre in Moshi, Tanzania. Women received a reminder-SMS 30 minutes before usual time of intake. One hour later, they received an SMS asking whether they took medication to which they could reply with ‘Yes’ or ‘No’. Messages were sent 3 times a week on randomly chosen days to prevent reliance on daily messages. We calculated the percentage of number of SMS delivered, failed to be delivered, and replied to. We analysed feedback from exit-interviews about experience with the SMS-reminders.

**Results:**

25 women were enrolled (age 18-45), 2 were lost to follow up. 5,054 messages were sent of which 53 failed to be delivered (1%). 1,880 SMS were sent with a question if medication was taken; 1,012 (54%) messages were replied to, of which 1,003 (99%) were replied with ‘YES’ and closely to ‘YES’, and a total of 9 (1%) with ‘NO’ and ‘closely to NO’. 868 messages (46%) were not responded to due to either dropout, change of phone number, loss of phone or network failure. Results from 18 interviews showed that 16 (89%) women were satisfied with SMS reminders. 2 (11%) were concerned about unwanted disclosure because of the content ‘don't forget to take medication’ and one reported other privacy issues (6%). 3 (17%) women experienced stigma.

**Conclusion:**

99%of SMS being delivered indicates that SMS reminders in this resource-limited setting are technically feasible. However, concerns regarding privacy were noted, specifically the risk of unwanted disclosure and the experience of stigma. Participants indicated that being made aware of their adherence, motivated them to adhere better. However, personalised and more neutral content of the SMS might be a way to improving the intervention.

## BACKGROUND

Infants are still being born with HIV in Sub Saharan Africa (SSA), despite significant increases in treatment coverage and implementation of programs to reduce vertical transmission of HIV.^1^ In East African countries, the coverage of Prevention of Mother-To-Child Transmission (PMTCT) programmes varied between countries from for example 77% in Kenya to 95% in Uganda in 2016.^2,3^ In Tanzania, there were 77,200 pregnant women living with HIV (WLHIV) in 2016.^4^ Of them, only 84% received free effective Antiretroviral Treatment (ART), resulting in a high mother-to-child transmission rate of 11%. The transmission rates of HIV from mother to child during pregnancy, delivery and breastfeeding vary from 15% to 45% in the absence of PMTCT programmes. According to the World Health Organization (WHO) guidelines, initiation of lifetime Antiretroviral Therapy (ART) by WLHIV under the recommended Option B+ programme has the potential to reduce the transmission of HIV to the newborn to below 5%.^6^ In addition, their infants should receive nevirapine syrup till 6 weeks postpartum and exclusive breastfeeding up to month 6, preferably continuing breastfeeding up to 24 months in addition to solid foods.^7^ In a prospective cohort study conducted in the Kilimanjaro region in 2016, out of 200 pregnant womenenrolled, 4.8% were found to be HIV positive while only 41% were in PMTC care.^5^ Sustaining a high level of adherence to ART during pregnancy, postpartum and during breastfeeding are, however, a prerequisite to prevent HIV-transmission from mother to child.^8^

Achieving optimal levels of adherence (>95%) is still a major challenge due to several factors including drug shortages and forgetting to take medication.^9^ Adherence to ART entails that medication is taken at the right time and exactly as prescribed without missing a dose. Poor adherence to ART may not only lead to virological failure and HIV-transmission from mother to child but also to creation of resistant HIV strains.

A meta-analysis among a large sample of People Living with HIV (PLHIV) outlined that worrying about disclosing the HIV status and forgetting to take medication on time were major barriers to adherence in Sub Saharan African countries.^10^ Achieving and maintaining high levels of adherence to ART is particularly challenging for pregnant and breast-feeding women. It was shown that pregnant women using ARV tend to forget taking doses of ART more often than non-pregnant women. They also may have more difficulties in incorporating medication intake into their busy schedule than non-pregnant women.^11^ Furthermore, it was shown that pregnant women may quit taking medication due to side-effects of ARV. Breastfeeding women may also feel more healthy after delivery leading to reduced motivation to continue taking medication.^1^

Nowadays, more than 80% of the population in Tanzania has access to mobile phones.^12^ Out of those, 60% own a basic phone without internet access and 20% a smart-phone. Short Message Service (SMS) has emerged as one of the leading mobile services.^12^ This provides a potential platform to support HIV treatment adherence by sending reminder cues through texting.

Several studies conducted in resource-limited settings examined the potential of mobile phone use in enhancing adherence to HIV medication. In a study conducted in Kwazulu Natal, South Africa, participants received an SMS once a week to remind them to take their HIV medication.^13,14^ A total of 98% of the participants reported that the SMS helped them to remember taking their medication. Two Randomised Clinical Trials (RCT) undertaken in Kenya indicated that weekly SMS reminders led to improved ART adherence.^15,16^ In Botswana, the adoption of SMS reminders has improved adherence to ART and also the relationship between patients and health care providers. In addition, results from that study showed that 93% of participants responded to the SMS reminders indicating it had helped them to take medication on time.^17^ WL-HIV were satisfied about SMS reminders as indicated in both studies in South Africa and Kenya.^13,15^ A 6 month pilot study conducted in South India, compared 2 to 3 times weekly SMS reminders with reminders via Interactive Voice Response (IVR). Adherence improved in both groups from 85% to 91%. However, all enrolled study participants would prefer automated IVR.^18^

Although previous studies showed that it is technically feasible to enhance adherence by sending SMS reminders and that this improved self-reported adherence, several challenges remain. In the studies in South Africa and Kenya, 10% of sent messages did not reach study participants due to loss of their phones and change of phone numbers during the study period. Also, they reported that participants who received daily SMS texts responded less often to these messages compared to participants who received weekly SMS texts.^15^ Participants reported concerns about privacy in studies in Kenya, South Africa and Botswana.^14,15,17^ Drop-out rates as high as 35% were reported in an RCT in Uganda.^19^ In this RCT, the reminder and a question asking about adherence were included in a single SMS. This combined question was not found to motivate participants to take their medication. Language illiteracy was noted to be a challenge in studies in Cameroon^20^ and Uganda^19^ as participants preferred SMS text messages in their native language instead of English. A systematic review of 35 studies about SMS applications in Africa found that only 5 studies evaluated the level of acceptance through exit interviews, showing that 94% of participants were highly satisfied. This review also indicated that network failure was a concern for 14% of enrolled participants causing them to sometimes miss the reminder SMS. Battery power was a problem to participants as well.^21^

Whereas SMS reminders are a promising method to enhance adherence to ART, challenges remain with respect to technical feasibility, acceptability, timing and content of the messages. In the present pilot study, we aim to investigate the acceptability and technical feasibility of using short text messages for enhancing adherence to ART among pregnant and breastfeeding WLHIV in Kilimanjaro, Tanzania.

## METHODS

### Study Design

This was a prospective, single-arm, 6-months observational pilot-study among HIV-positive pregnant and breastfeeding women. The study was approved by the Kilimanjaro Christian Medical College Research Ethics and Review Committee (CRERC) No.829 and the National Health Research Ethics Sub-Committee (NathRec) of Tanzania NIMR/HQ/R.8a/Vol.1X/2432.

### Study Participants

From May 2017 to July 2017, we recruited pregnant and breastfeeding women who were attending either ante-natal or postnatal care at Kilimanjaro Christian Medical Center (KCMC) in Moshi, Tanzania. KCMC is a tertiary referral hospital in the Northern zone of Tanzania with 450 beds and provides service to approximately 250 to 300 pregnant and breastfeeding WLHIV annually. The centre includes a special Child Centred Family Care Clinic (CCFCC), which provides care and treatment to children living with HIV/AIDS and their families. Women were eligible for the study if they (1) were HIV positive and pregnant or breastfeeding, (2) were aged between 18 and 50 years, (3) were attending Kilimanjaro Christian Medical Centre, (4) were on ART since at least 6 months, (5) had no foreseen changes in ART in the subsequent 3 months, (6) owned a mobile phone with operational SIM card, (7) lived in rural or urban areas of the Kilimanjaro Region, (8) were willing to receive SMS reminding them to take ART, (9) were able to read and reply to SMS, (10) were willing to come to the clinic at least once a month and (11) provided written informed consent to participate in the study. We excluded WLHIV who were either on co-medication for other (chronic) diseases such as tuberculosis (TB), diabetes and chronic hypertension; or who were admitted to a hospital or were participating in concurrent SMS reminder studies. As this was a pilot study investigating the feasibility of a newly developed intervention, a sample size of 25 WLHIV was considered sufficient to meet our aims.

### Study Procedures Recruitment and Monitoring of WLHIV

We used convenience sampling to recruit women. Potential participants were identified by a clinic nurse who informed the research doctor. The doctor determined whether a candidate fulfilled the eligibility criteria. The study nurses explained the study in detail to the eligible participant using a participant information leaflet. The participant was given time to read and understand the leaflet. Written informed consent was requested from participants before any study procedure. Following that, the nurse invited all women who agreed to participate. Subsequently, baseline information about the participants was collected through a short structured questionnaire asking about demographics, mobile phone number and usual time of medication intake according to the physician's prescription. We entered the participant's phone number into the SMS program and subscribed the participant's phone number to an unlimited monthly SMS bundle.

After enrolment, participants were followed for 6 months. The participants attended the clinic monthly for medication refill and antenatal follow-up. After 3 months, the study doctor asked a general question “*What is your general experience with the SMS messages?”* The answer was written down verbatim.

After 6 months, participants were interviewed about their experience with receiving SMS by using a semi-structured questionnaire. The interview was conducted in Swahili by the study doctor. Data from the enrolment questionnaire and the exit-interviews were entered in REDCap® (Research Electronic Data Capture) 9.3.5, Vanderbilt University, Tennessee USA, an open-source web-based system which has features for query generating, auditing and data validation.^22^

### The Intervention

#### SMS Content

**FIGURE 1.**
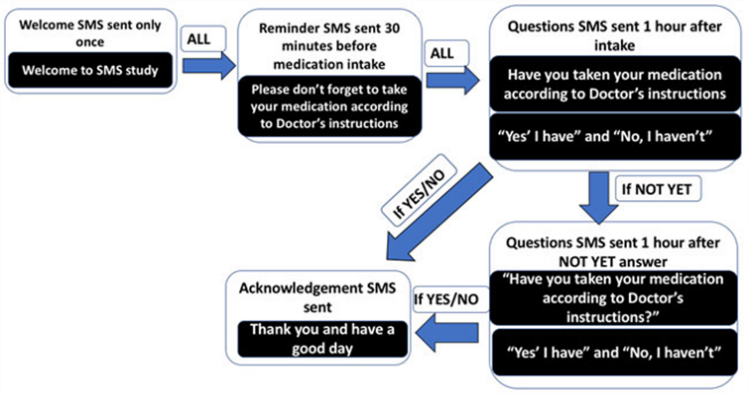
Flow of SMS Messages Sent to Participants

The SMS were grouped into 4 categories. We designed the SMS in Swahili and only those were sent to our participants. Figure 1 shows the SMS scheme translated into English for the purpose of general readability of this manuscript. First, an introduction SMS was sent once on the day of enrolment to welcome the participant. Second, a reminder message to alert the participant to not forget to take their medication was sent 30 minutes before usual time of medication intake. Thirdly, a question SMS was sent one hour after usual time of intake to ask the participants if they took the medication according to the doctor's instruction. The participant had to reply with any of the options ‘N’ (Ndiyo, meaning YES) - I took my medication, ‘H’ (Hapana, meaning NO) - I did not take my medication or ‘B’ (Bado, meaning NOT YET). If the reply was YES or NO, an acknowledgement SMS was sent saying “Thank you and have good day” and the SMS flow was terminated. If the reply was “NOT YET”, one hour later, a question SMS was sent again to ask if medication was taken.

The timing of the SMS reminders was scheduled individually and processed automatically by the SMS system. The SMS system sent messages 3 times a week on randomly chosen days. The days were different for each participant. We used the permutations formula:

$$\begin{array}{l} \text{n}!=\\ {}_{\text{n}}P_{\text{r}}=(\text{n}-\text{r})! \end{array}$$

for calculation of the number of possible combinations of 3 days in a week of 7 days. 35 combinations of days were possible (i.e. Mon-Tues-Wed, Mon-Tues-Thurs, Mon-Tues-Fri, Mon-Tues-Sat etc.).

### The SMS Program

The SMS program was developed using open-source software Telerivet® (San Francisco, California, United States).^23^ The software has a standard platform that integrates most of the existing mobile phone technologies. Telerivet allows routing of messages to and from any number of mobile devices with a basic internet connection. With a cloud-based management system, it supports the developer to adapt an external Application Platform Interface (API) using other platforms for monitoring and tracking activities. The system is only accessible through password authorisation.

### Keywords

Keywords are pre-defined words that women could respond to by typing an SMS reply. Oncethe SMS from a participant is received, the SMS program scans the message content and matches it with our pre-defined keywords such as ‘YES’ and ‘NO’. When keywords were recognised, the program automatically sent a reply to the participant based on the specified conditions and algorithm that we programmed in the system.

### Outcome measures Technical Feasibility of the SMS Program

Technical feasibility was based on the degree of performance and success of the system operation. Performance was determined by tracking the total number of SMS messages sent and delivered per participant; specifically, the number of SMS reminders and SMS questions. The success of the SMS system was based on scheduling of SMS messages, which includes the time of intake and date of SMS sent, failed and delivered. The outcome of feasibility was measured through calculating percentages. The numerator was the total number of SMS delivered and the denominator was the total number of SMS sent.

### Acceptability of Receiving SMS Reminders and Questions

Acceptability was evaluated in terms of satisfaction with receiving SMS reminders and questions by participants. This was evaluated using a questionnaire containing 18 questions. Each question had closed-ended response options to be answered by “yes” or “no” or “good” or not “good” followed by open-ended questions to solicit explanations. The open-ended questions provided more narrative explanations of the answers selected in the closed questions. The questionnaire was developed by the study team based on feedback given during consultations and on previous research.^16^ The topics were on general experience with receiving the SMS reminders and questions, difficulties in receiving SMS, timing of SMS, contents of the SMS, problems with network connectivity, travelling, advantages of the SMS, potential stigma and loss of confidentiality by receiving SMS, ability to reply to SMS, impact on adherence, taking medication on days without SMS and ideas about adherence promoting interventions. Descriptive analyses (frequencies and percentages) of the outcomes were conducted to measure the acceptability. The numerator was the frequency calculated from closed-ended question. The denominator was the total number of participants that participated in the exit-interview.

### Adherence to Medication

To obtain an indication about the extent to which SMS can be used for adherence monitoring, we calculated adherence based on SMS replies. ‘Yes’ answers were seen as an indication (“proxy”) for medication intake. We calculated an adherence percentage for each participant based on the number of responses that contained the keyword ‘YES’ or similar to ‘YES’ and ‘NO’ or similar to ‘NO’ (numerator). This was divided by the total number of question SMS sent to the participants one hour after usual time of medication intake (denominator). SMS with responses similar to ‘YES’ were ‘I TOOK MY PILLS’, ‘I REMEMBERED TO TAKE MY PILLS’, ‘THANKS FOR REMINDING ME’. These responses were considered “similar to Yes” and we assumed that these responses indicated that women took their medication. The responses determined to be similar to ‘NO’ where ‘I DIDN’T TAKE IT’, ‘I FORGOT TO TAKE IT’ and ‘I COULD NOT TAKE IT’.

### Data Analyses

We used IBM SPSS software® version 24 (New York, US) for statistical analyses to determine the frequencies of answers to the SMS messages. Responses to the questions of the exit-interview were presented as frequencies and percentages. Narratives from feedback during consultation and from the exit-interviews were used to illustrate the frequencies. We calculated adherence based on the SMS messages (‘YES’ meaning medication was taken) as stated in the previous paragraph for each woman who participated. From there we calculated median adherence for all included women.

## RESULTS

25 women participated in our study. Their overall characteristics and variables related to HIV are shown in Table 1. 2 women dropped out before the end of the study and could not be traced for the exit interview. Data for all 25 participants are included in Table 1. 8 women (32%) were pregnant at the time of inclusion and 17(68%) were breastfeeding. The median duration on ART was 4 years (range: 0.5-12) and median age of participants was 36 (range 23-43)]. 22 (88%) women were on first-line ART and 3 (12%) were on second-line ART. (Table 1)

**TABLE 1:** Characteristics of Pregnant and Breast-Feeding Women (N=25)

Characteristics	(%) or Median [IQR^*^]
Pregnant women	8 (32%)
Breastfeeding women	17 (68%)
Age (Years)	10[range 30–40]
Duration on ART before the study (years)	2 [range: 6–8]
On frst line ART (TLE) (DUOVIR-N)	22 (88%)
On second line ART (LPV/r + TDF +FTC) OR (ATV/r + ABC + 3TC)	3 (12%)

TLE: Tenofovir+Lamivudine+Efavirenz ATV/r + ABC + 3TC: Atazanavir/Ritonavir (ATV/r) + Abacavir/Lamivudine (ABC/3TC) LPV/r + TDF +FTC: Tenofovir +Emtricitabine (Truvada) + Lopina-vir-ritonavir (LPV/r)

* IQR=Interquartile Range

### Technical Feasibility of Sending and Receiving Messages

In total, 5,054 SMS were scheduled and sent of which 5,001 (99%) were delivered. 53 (1%) were not delivered. (Table 2). 4 participants occasionally received SMS messages too late despite having been sent at the scheduled time, which appeared to be due to participants switching off their phone during the night or network failures. SMS messages were resent the next day. A total of 1,845 (99.6% of total sent) SMS reminders were delivered 30 minutes before medication intake and 1,880 (99.5% of total sent) SMS questions were delivered one hour after scheduled intake to ask the participants whether they took their medication or not. More question SMSs were sent than reminders, since the question was repeated after one hour if the reply was ‘Not yet’. (Table 2)

**TABLE 2:** SMS Overview

Variables	Number of SMS (%)
Total SMS scheduled and sent for 25 participants	5054
• Delivered	5001 (99%)
• Failed	53 (1%)
Reminder SMS that were delivered “Please don't forget to take your medication according to Doctor's instruction”	1845 (99.6%)
Question SMS that were delivered “Have you taken your medication according to Doctor's instructions?”	1880 (99.5%)*

### Acceptability of SMS Reminders and Questions

18 (72%) participants were reached and willing to participate in the exit interviews whereas 5 participants could not be reached for follow-up (Table 4). 2 of 25 participants asked to be removed from the study before the end of the 6 months follow up due to the SMS content mentioning the word “medication”. These participants proposed the SMS content to contain neutral or customised words. Other participants also mentioned the concern of limited confidentiality which might lead to disclosure of their HIV status. This was explained by one participant saying *“For instance the reminder question ‘Did you take your medication’ may lead to lack of confidentiality or disclosure of my status to my friends or partner”*. Another comment was *“The SMS as they come in, sometimes I am not with my phone, therefore I'm worried someone else could see that SMS”*. Most participants (75%) reported to be satisfied with receiving SMS reminders and questions. For example, one participant acknowledged that *“the reminder SMS was very supportive to me to remind me to take medication because several times I am busy with my usual activities”*. Others described the desire for continuing to receive reminder messages after the end of the study. 4 of the 18 participants (22%) expressed having difficulties in receiving the reminder SMS. The feedbacks from those participants were*, “The SMS were coming late, most of the times about 30 minutes later”, “Sometimes I find the SMS the next morning”, “Sometimes the SMS delays up to one day”*. (Table 4)

**TABLE 3:** Overview of SMS Sent and Delivered Per Woman.

		Before cleaning Yes						After cleaning							
Number	Reminder	Question	Yes	No	TOTAL	%answered	Adherence YES/Message SENT	Adherence based on answers	Question	Yes	No	TOTAL ANSWERS	%answered	Adherence YES/Message SENT	Adherence
587	36	35	26	1	27	77%	74%	96%	35	26	1	27	77%	74%	96%
349	77	79	67	0	67	85%	85%	100%	79	67	0	67	85%	85%	100%
506	78	78	5	0	5	6%	6%	100%	78	67	0	67	86%	86%	100%
812	78	81	28	0	28	35%	35%	100%	81	28	0	28	35%	35%	100%
561	78	83	16	11	7	20%	19%	94%	83	17	1	18	22%	20%	94%
046	78	79	5	0	5	6%	6%	100%	79	5	0	5	6%	6%	100%
941	78	83	46	0	46	55%	55%	100%	83	48	0	48	58%	58%	100%
001	78	78	46	1	47	60%	59%	98%	78	46	1	47	60%	59%	98%
170	78	78	43	0	43	55%	55%	100%	78	44	0	44	56%	56%	100%
126	78	80	64	2	66	83%	80%	97%	80	64	2	66	83%	80%	97%
414	78	87	7	0	7	8%	8%	100%	87	60	0	60	69%	69%	100%
266	81	81	5	0	5	6%	6%	100%	81	17	0	17	21%	21%	100%
028	78	78	36	0	36	46%	46%	100%	78	66	0	66	85%	85%	100%
086	78	78	27	0	27	35%	35%	100%	78	27	0	27	35%	35%	100%
446	78	78	42	0	42	54%	54%	100%	78	43	0	43	55%	55%	100%
556	78	79	40	2	42	53%	51%	95%	79	41	2	43	54%	52%	95%
911	78	78	57	0	57	73%	73%	100%	78	57	0	57	73%	73%	100%
299	78	78	51	2	53	68%	65%	96%	78	51	2	53	68%	65%	96%
221	78	79	54	0	54	68%	68%	100%	79	54	0	54	68%	68%	100%
179	78	78	44	0	44	56%	56%	100%	78	52	0	52	67%	67%	100%
195	78	78	0	0	0	0%	0%		78	0	0	0	0%	0%	
977	78	82	12	0	12	15%	15%	100%	82	17	0	17	21%	21%	100%
455	13	13	0	0	0	0%	0%		13	0	0	0	0%	0%	
248	78	80	30	0	30	38%	38%	100%	80	37	0	37	46%	46%	100%
811	78	79	67	0	67	85%	85%	100%	79	69	0	69	87%	87%	100%
**TOTAL**	**1845**	**1880**	**818**	**9**	**827**	**44%**	**44%**	**99%**	**1880**	**1003**	**9**	**1012**	**54%**	**53%**	**99%**
**MEDIAI**							53%	51%	100%				58%	58%	100%
**MEAN**							44%	43%	99%				53%	52%	99%

^*^before cleaning: analysis included only the keyword “YES, NO” ^*^after cleaning” analysis included keyword “YES, NO, Similar with YES and Similar with NO ”

**TABLE 4:** Feedback on Receiving SMS Reminders and Messages

Participants feedback	N 18 (72%)
General experience with receiving SMS	
Not good at all	1 (5.6)
Not good	1 (5.6)
Good	2 (11.1)
Very good	14 (77.8)
Experience with SMS system not good at all (n=2)	
Lack of confdentiality – disclosure	
I don't like, it is not safe on my side	
SMS came on time	
Yes	15 (83.3)
No	3 (16.7)
SMS did not come on time (n=3)	
SMS delivered 30mins later	
SMS delivered on the following morning	
SMS delivered one day later	
Diffculties with receiving SMS	
Yes	4 (22.2)
No	14 (77.8)
Diffculties (n=4)	
Some days there were no SMS received	
Delay in receiving SMS	
Missing	
Opinion about content	
Not good at all	3 (16.7)
Not good	1 (5.6)
Good	2 (11.1)
Very good	12 (66.7)
Content is not good or not good at all (n=4)	
The words ‘you are reminded to take medication’ was not good to me	
It breaks the confdentiality	
The word ‘kumeza dawa’ (take medication) is not good. It breaches confdentiality	
The SMS which say ‘kumeza dawa (take medication)’ is a bit not good if someone else sees it	

### Adherence to Responding to SMS Questions

Out of 1,880 SMS sent with the question “Did you take medication?”, a total of 1,012 (54%) were replied to. 818 (44%) SMS were replied to with the keyword ‘YES’ and a total of 185 (10%) were replied to with similar to-‘YES’ indicating that medication was taken. A total of 864 SMS (46%) were not replied to. 9 SMS (1%) replied with ‘NO’ and similar to ‘NO’ (see table 5). The median adherence based on YES-replies was 51% (range 0-85). The median adherence based on YES and similar-to-YES-replies was 58% (range 0-87). (Table 5)

**TABLE 5:** Adherence Based on SMS Replies

Variables	Number of SMS (%) or Median [IQR]
Total SMS sent with question “Have you taken your medication according to Doctor's instructions”	1880
Total Replied (YES, closely to YES, NO, closely to NO, NOT YET)	1012 (54%)
• Replied with YES	818 (81%)
• Total Replied YES and Closely to YES	1003 (99%)
• Replied with NO or closely to NO	9 (<1%)
Total not replied	864 (46%)

## DISCUSSION

In this pilot study, we investigated the acceptability and feasibility of sending SMS reminders to pregnant and breastfeeding WLHIV in Kilimanjaro (Tanzania) to take their antiretroviral medication. Almost all SMS that were sent, i.e. 99%, were actually delivered, supporting its technical feasibility. 54% of all monitoring SMS were replied to. The majority of participants found it acceptable to receive SMS that reminded them to take their medication and were satisfied with the content. However, there were also participants who expressed concerns about their privacy and were afraid that receiving the SMS could disclose their HIV status to others.

Only 1% of SMS was not delivered. Although we do not have data about each individual reason, they are most likely related to network failure, loss of phone and change of phone number. The finding that 46% of the question-SMS were not replied to was unexpected and disappointing. We were uncertain about the underlying reasons. Our primary thought was that participants did not understand or were annoyed by the messages. Also, there could have been changes of phone numbers, network failures, or participants could have been non-adherent. In the exit-interviews, the main reasons mentioned by participants were sharing of phones and network issue. Also, the adherence percentage of 58% is rather low, but as this was based on ‘YES’-replies only this rather represents adherence to the SMS. In general, studies have shown that the mean adherence to ART among pregnant women in sub-Saharan Africa countries ranges from 35% to 93.5%.^24^ For instance, a study in the Eastern Cape, South Africa showed an adherence level of 69% among pregnant women.^25^ Some participants mentioned that they did not have an SMS bundle enabling them to reply, despite the fact that we sent them a monthly bundle. The fact that a few participants experienced stigma and had disclosure concerns, was expected. This finding is consistent with studies in South Africa, Kenya and Uganda that showed that some participants had concerns about the privacy of SMS^14,26,27^ 2 participants withdrew consent during follow-up and 5 others could not be traced anymore at the end of the study. This is a common problem in resource-limited settings where there are challenges of retaining pregnant and breastfeeding women in care.^24,28,29^ Many pregnant women discover their positive HIV status during pregnancy and are worried to disclose their status to their husbands or others.^27^

Our findings are in line with other studies showing that receiving SMS reminders might be intrusive. For example, Rashmi et al. in South India have recommended that weekly SMS should be sent at a time that is convenient for participants to reduce intrusion.^11^ In this study, participants indicated that being aware that their adherence to medication was monitored, motivated them to adhere better. Similar to our study, participants mentioned that knowing someone caring for them by reminding them of the intakes, helped them to be adherent. The study of Mushamiri et al ^30^ found high response rates to reminder and question SMS in the first 3 months, but a clear decline in response rates thereafter. This suggests that participants may get used to SMS messages and ignore responding to the text despite being adherent.

This study has several limitations. First, the focus of the study was on technical feasibility and experience of users which both showed positive results. This means we cannot draw any formal conclusions regarding adherence to treatment. Also, the level of adherence was based on the reply-SMSs, which is indirect and self-reported. We are not sure if the percentages of ‘Yes’-answers are a true representation of adherence, as we did not measure accuracy through pill counts, questionnaires, direct measurements of drug concentrations, or with virological outcome. Despiteour study design not being optimal to relate “Yes responses” to true drug adherence, the study may provide some indication of self-reported adherence. As such SMS may, to a certain extent allow monitoring of adherence, although adaptations to the program is likely needed. Another limitation is that only women who own a mobile phone were enrolled. This means that WLHIV without a mobile phone were excluded. Therefore, the results cannot be generalised to WLHIV without a phone, who may have different characteristics such as living in rural areas, low socio-economic status and high illiteracy. In future studies, mobile phones may be supplied by adherence programmes.

One strength of our study is that it included so called interactive 2-way text messaging which encompasses receiving replies from participants and automated SMS sent back to participants. Another strength is that we were able to send the SMS content in local language that is understood and spoken by the vast majority of participants in contrast to several previous studies. Furthermore, the costs of the SMS program in terms of infrastructure investment were low. The sending of automated reminder SMS to 25 participants cost less than 2 US dollars per month. Also, being automated, we managed to decrease the burden on nurses by not having to involve them to send SMS to participants.

Based on our study, we recommend examining whether more neutral messages will trigger medication intake while not disclosing the HIV status. For instance, instead of the standard message ‘Did you take your medication’, we could ask ‘did you do as recommended?’ Furthermore, it is important to investigate whether the SMS program will really improve adherence in our setting. Therefore, clinical trials are needed to investigate the effect on adherence and implementation studies are needed to examine the effectiveness of the program in a real-world setting. WLHIV who will use the SMS program, whether it is in the context of a study or as part of regular care, should receive proper explanation about the SMS and its contents. Only in that way, they can make a well-informed decision whether to make use of the SMS reminders. WLHIV who are worried about unwanted disclosure can decline using it.

## CONCLUSION

We found that it is technically feasible and acceptable to the majority of pregnant and breastfeeding women living with HIV to receive SMS to remind them to take their medication. There were a few women who expressed concerns about their privacy and were afraid that receiving the messages could lead to unwanted disclosure of their HIV status. Understanding and addressing such potential barriers and challenges will be crucial for researchers and policy makers to improve the design of future studies involving SMS and their successful implementation in practice. Moreover, further investigation of the impact of SMS on adherence to ART in other groups living with HIV is recommended.
